# The roles of self efficacy and sharia financial literacy to SMES performance: business model as intermediate variable

**DOI:** 10.12688/f1000research.76001.1

**Published:** 2021-12-22

**Authors:** Popon Srisusilawati, Zaini Abdul Malik, Irma Yulita Silviany, Nanik Eprianti

**Affiliations:** 1 Sharia Faculty, Universitas Islam Bandung, Bandung, Jawa Barat, 40116, Indonesia

**Keywords:** Self-efficacy, literacy Islamic finance, Innovation business model, The performance of SMEs

## Abstract

**Background**: SMEs have made a major contribution to the national economy, but Pandemic covid-19 made the decline of SMEs which directly affected the economy in Indonesia. The purpose of this research focus to explore the innovation of business models the novelty of the research model in the business model of innovation research models is to include the variable self-efficacy. This research emphasizes self-efficacy and Islamic financial literacy as independent variables, business model innovation as an intermediate model and business performance variables as dependent variables, with the focus of research on SMEs. **Methods**: This quantitative study uses partial least square structural equation modeling (PLS-SEM) and a questionnaire survey to analyze the 40 Street Vendors which is categorized as SMEs around Unisba. In the initial stages, the Questionnaire was tested with the Validity and Reliability test. In the PLS-SEM analysis, the validity of the indicators / items forming latent variables is based on the significance value of t arithmetic obtained from the loading factor value divided by the standard error. After being collected, the data is then processed and analyzed. The analytical method used in this study is the simultaneous equation model, namely Partial Least Square (PLS). The statistical tool used to test the hypothesis is Smart PLS. **Results**: The results showed that self-efficacy and Islamic financial literacy through business model innovation, had a positive effect on SMEs business performance simultaneously or partially. **Conclusion**: So that by having good self-efficacy and good Islamic financial literacy plus an appropriate business innovation model, it will improve the business performance of SMEs.

## Introduction

Small Medium Enterprises (SMEs) play a very important role in economic growth, in both developing and developed countries. In developing countries the perspective of employment opportunities and sources of income for the poor, income distribution and poverty reduction and rural economic development ( Gura, no date; Chrismardani, 2014).

The Ministry of Cooperatives and Small and Medium Enterprises (KemenkopUKM) shows that in 2018 there were 64,194,057 SMEs in Indonesia (or around 99 percent of the total business units) ( Pakpahan, 2020). The development of SMEs in Indonesia is inseparable from the factors that drive growth, technological utilization, communication and business capital lending ( Hani, Mariati and Harahap, 2018). However, the growth is still considered slow because several factors have not been very effective, one of them is capital ( Ekonomi and Surabaya, 2016). Amid the development of SMEs that were not very good at the beginning of 2020, SMEs in Indonesia were again tested for the emergence of the Covid-19 outbreak amid Indonesian society. Rising the Covid-19 pandemic the government imposed large-scale social restrictions (PSBB) ( Jokowi Terapkan Social Distancing Atasi Wabah Corona Covid-19, Apa Artinya?, no date). These efforts have an impact on the drastic decline in SMEs, impeded production, distribution and sales that contribute to SMEs performance and the national economy ( Pakpahan, 2020).

Based on this phenomenon, SMEs are facing rapid changes resulting in the current business model is no longer sustainable. The driving force for changes in the SMEs industry - self-efficacy and Sharia financial literacy - is increasing because these facts are interrelated ( Fatah Yasin, Mahmud and Diniyya, 2020). Self-efficacy is needed by every resource in order to be able to face changes so as to encourage innovation in business models ( Putri *et al.*, 2019), strengthening Shariah financial literacy can improve business performance ( Hidajat *et al.*, 2016). So, SMEs need to sharpen their strategies so that they can remain relevant in the Covid-19 pandemic.

The Covid-19 pandemic phenomenon causes the problem of decreasing assets, SMEs market share in Indonesia ( Shofiana, 2020; Kholis, Fraternesi and Wahidin, 2020). This phenomenon can be identified as a Business Performance variable. The phenomenon of SMEs to integrate, build and reconfigure competencies to cope with a rapidly changing environment ( Coff, 2003; Kim and Mahoney, 2005), is identified as a variable of self-efficacy. The phenomenon of the influence of buyer preferences on the purchase of products and services so that SMEs must have sufficient knowledge of market needs can be identified as a resource variable. The phenomenon of capital towards managers in developing sharia SMEs products and services is identified as a sharia financial literacy variable. The phenomenon of SMEs to make changes to important business processes, the value offered to the market and the changes identified as innovation business models.

This research emphasizes self-efficacy, Islamic financial literacy as independent variables, business model innovation as an intermediate model and performance variables as dependent variables, with a focus of research at the SMEs level in Indonesia, so that the aspects of concern are dominant aspects affecting business model innovation and SMEs performance. This study assumes two main variables that influence or become determinants of the innovation of the SMEs business model. So the purpose of this research is whether it influences or determines the innovation of the SMEs business model.

### Theoretical basis

The variables in this study are self-efficacy (SE), Islamic financial literacy, business model innovation and performance which are built based on the Resource Based View (RBV) theory where the RBV concept according to ( Barney, 1991) manages the potential resources of the company to achieve long-term competitive advantage.

So that the research examines self-efficacy, Islamic financial literacy, its influence on the performance of SMEs by moderating business model innovation. Several previous studies related to the relationship of the variables studied contained contradictions or differences in research results, so that it became a research gap that could be studied further from the limitations of previous research.

Bandura (2010) revealed that self-efficacy is an assessment of self-confidence about how well individuals can perform the necessary actions related to prospective situations ( Singh and Padhi, 2019) stated that the role of self-efficacy has a positive effect on performance ( Sebayang and Sembiring, 2017) agree that self-efficacy has a significant effect on employee performance. So that it examines self-efficacy, Islamic financial literacy, its influence on the performance of MSMEs by moderating business innovation models. Several previous studies related to the relationship of the variables studied contained contradictions or differences in research results, so that it became a research gap that could be studied further from the limitations of previous research. With regard to motivation and performance, Bandura and Locke ( Bandura and Locke, 2003) found that self-efficacy and self- goals can improve these aspects, while Ali ( Ali, Ajmal and Iqbal, 2016) shows that self-efficacy can improve orientation entrepreneurship and decision making. Both of these results are in line with Bandura ( Bandura, 2010) which states that self-efficacy influences individual behavior, motivation, success, and failure. In contrast, Vancouver, Thompson, and Williams ( Vancouver *et al.*, 2002; Schmidt and DeShon, 2010) find that self-efficacy has no impact on business performance. Based on these findings, there is a gap in the literature about the impact of self-efficacy on business performance.

In addition to self-efficacy, SMEs face certain difficulties, due to the limited financial literacy of their owners. According to Lusardi and Mitchell ( Lusardi and Mitchell, 2017) financial literacy refers to financial knowledge used to achieve prosperity, while Chen and Volpe ( Volpe, Chen and Liu, 2006) define this term as the knowledge used to manage finances for future prosperity. In addition, several studies on the importance of financial literacy on business performance in business continuity ( ISLAM, no date; Rahayu *et al.*, no date; Suryandani, Nasional and 2019, no date; Anggraeni, 2016; Aribawa, 2016; Literasi *et al.*, 2020). However, based on Badrusoleh ( SHOLEH, 2019) the community has a low level of financial literacy. In this case, Tamvada ( Coad and Tamvada, 2012) shows that lack of knowledge and access to financial resources can hamper a company's ability to achieve its goals and increase its corporate value. Even Djuwita and Yusup ( Djuwita and Yusuf, 2018) in their research stated that Shariah financial literacy has a significant influence on business development. This will facilitate access to capital and financing to Islamic financial institutions. Therefore, this study fills the gap between conventional financial literacy and Islamic financial literacy, because the two types have different goals. Based on research ( Lembaga Keuangan *et al.*, 2021) that Islamic financial literacy has a positive and significant effect on the performance of small businesses. Furthermore, according to ( Woli and Wardani, 2021) that self-efficacy and Islamic financial literacy affect interest and performance.

According to ( Rahim, Reskillah and Nasfi, 2020) Islamic financial literacy is an extension of financial literacy with sharia-compliant elements. Conceptually, Islamic financial literacy is defined as a person's ability to use financial knowledge, skills and attitudes in managing financial resources according to Islamic teachings.

The relationship between socio-economic concerns and business success is not something that happens automatically. A business case for sustainability must be created ( Wagner, 2007). The consequence of the goal of implementing sustainable business is that management is challenged to find new approaches to realize potential business cases through adequate management of sustainability.

Theoretical considerations show that various approaches to integrate aspects of sustainability into the business model and also its expansion must be present and that this must be directly related to the extent to which environmental and social aspects have been embedded in the performance of companies that underlie the business model. In general, it seems clear that because environmental and social issues gain relevance in strategy, modifications to the broader business model must be made; both, regarding the modification of existing models and the development of new sketches of existing business models.

Two considerations that explain why business performance and business models are closely related. First, if companies implement strategies aimed at business cases that are sustainable, for example ( Schaltegger and Wagner, 2011) business models may have to change (directly or indirectly); that is, the need for triggers for a better business case (e.g., the need to improve the cost structure because of more expensive but environmentally friendly production inputs) may affect the configuration of the business model. Second, and vice versa, the business model also determines and limits business performance and business case sustainability. Business models are often interpreted as determinants of corporate behavior and thus business opportunities ( Aagaard, 2019; Chesbrough, 2010; Gebauer *et al.*, 2017; Schaltegger, Lüdeke-Freund and Hansen, 2011; Schaltegger and Wagner, 2011) yang menyebut model bisnis “pembaruan bisnis”; Wirtz, Yip, Zott dkk. ( Wirtz, 2011; Yip, 2014; Zott, Amit and Massa, 2011); that is, the business model in turn influences business performance and operating results (such as cost structures).

The results show that companies that are trying to improve their sustainability performance must change their business models. Changes in business models can be incremental or radical can turn into determinants (ie limit or support) to successfully create one or many business cases for sustainability (regarding the intensity of modification and innovation of different business models, see Chesbrough, Gassmann and Henry ( Chesbrough, 2010; Gassmann, Enkel and Chesbrough, 2010; Chesbrough, Vanhaverbeke and West, 2014).

Novelty in this study emphasizes self-efficacy, Sharia financial literacy as independent variables, sharia business model innovation as an intermediate model and performance variables as dependent variables, with a focus of research on SMEs in Indonesia, so that the aspects of concern are dominant aspects influencing business model innovation and SMES performance. This study assumes two main variables that influence or become determinants of the innovation of the SMES business model in a Covid-19 pandemic.

## Methods

This quantitative study uses partial least square structural equation modeling (PLS-SEM) and a questionnaire survey to analyze 40 SMEs especially row stalls that are managed by 3IKB (women association of Unisba) around Unisba. The research location was determined purposively, from January to April 2021.

The sampling technique used is saturated sampling, that is, all of the population is used as a sample, namely 40 SMEs at the location of the Unisba Row stall managed by 3IKB. The primary data used are measurement and data collection directly by researchers with questionnaires. Filling out the questionnaire was carried out by filling in directly by the research subject.

Instruments which is developed by Bandura ( Bandura, 2010) was used to measure self-efficacy whereas about instrument of financial literacy Syariah, consisting of indicators: business experience; Islamic Motivation; Islamic business training; and Islamic education Sharia financial management. In addition, instruments adapted from Djuwita and Yusup ( Djuwita and Yusuf, 2018) are used to measure Islamic financial literacy, while instruments from Aribawa ( Aribawa, 2016) are used to measure SMES performance, consisting of five dimensions: profitability; growth; employee satisfaction; customer satisfaction; and environmental performance. While. The last instrument of business model innovation according to Chesbrougt ( Chesbrough, Vanhaverbeke and West, 2014) consists of value creation innovation value proposition innovation value capture innovation.

In the initial stages, the Questionnaire was tested with the Validity and Reliability test. In the PLS-SEM analysis, the validity of the indicators/items forming latent variables is based on the significance value of t arithmetic obtained from the loading factor value divided by the standard error. Validity and reliability tests were conducted to measure the validity and reliability of the variables proposed by the researchers. Overall, the validity test determines whether the external loading of each indicator exceeds 0.4 (standard value), while the reliability test determines whether the value exceeds Cronbach's alpha 0,6, which shows acceptable reliability.

Evaluation of external models is done by examining two elements, namely: convergent validity and discriminant validity. In general, convergent validity is determined by examining the loading factor and extracted average variance (AVE), while discriminant validity is measured by examining cross-loading values. In this study, all factor loading meets the standard value of 0.4, while all AVE values exceed 0.5, thus indicating that convergent validity is achieved. Based on the inspection of cross-load values, the discriminant validity also increases. The model is evaluated according to R-square for endogenous latent variables and path coefficient values, the results of which are shown in Table 1

**Table 1.** R-square results.

	R-square
Self-efficacy	-
Sharia financial literacy	-
SME performance	0. 899

Source: Output partial least squares structural equation modeling (2020).

According to Table 1, the R-square for the SMEs performance is 0, 899, which shows that it is influenced by self-efficacy and Islamic financial literacy. The inner model is also measured through the predictive relevance of Q-square. Because Q-square is greater than 0, the model has predictive relevance for exogenous and endogenous variables.

After being collected, whereas instrument of financial. The analytical method used in this study is the simultaneous equation model, namely Partial Least Square (PLS). This method is also the development of path analysis. The statistical tool used to test the hypothesis is SmartPLS.

The process of processing statistical data with PLS is done by 2 approaches, namely (i) estimation of the measurement model and (ii) structural model estimation. In the measurement model test, the conformity test with indicators included Chi-Square, Root mean square residual (RMR), Roor Mean Square Error of Approxation (RMSEA), Adjusted good of fit index (AGFI), and so on. In structural model tests, the influence of exogenous latent variables to endogenous latent variables that are direct and indirect can be determined, as well as determining the direct and indirect effects of path analysis. The direct effect shows the direct effect of a variable on other variables. Indirect effects indicate the indirect effect of a variable on other variables through other variables. Total effect is the sum of direct effects and indirect effects.

## Result and discussion

This study focuses on the influence of self-efficacy (X1) and financial literacy sharia (X 2) at Innovation Business Model (Y) on the Performance of Business (Z) on SMEs in Unisba row stall Indomesia are analyzed based on research data using Analysis of Structural Equation Modeling (SEM) Smart PLS software tools.

**Figure 1.**
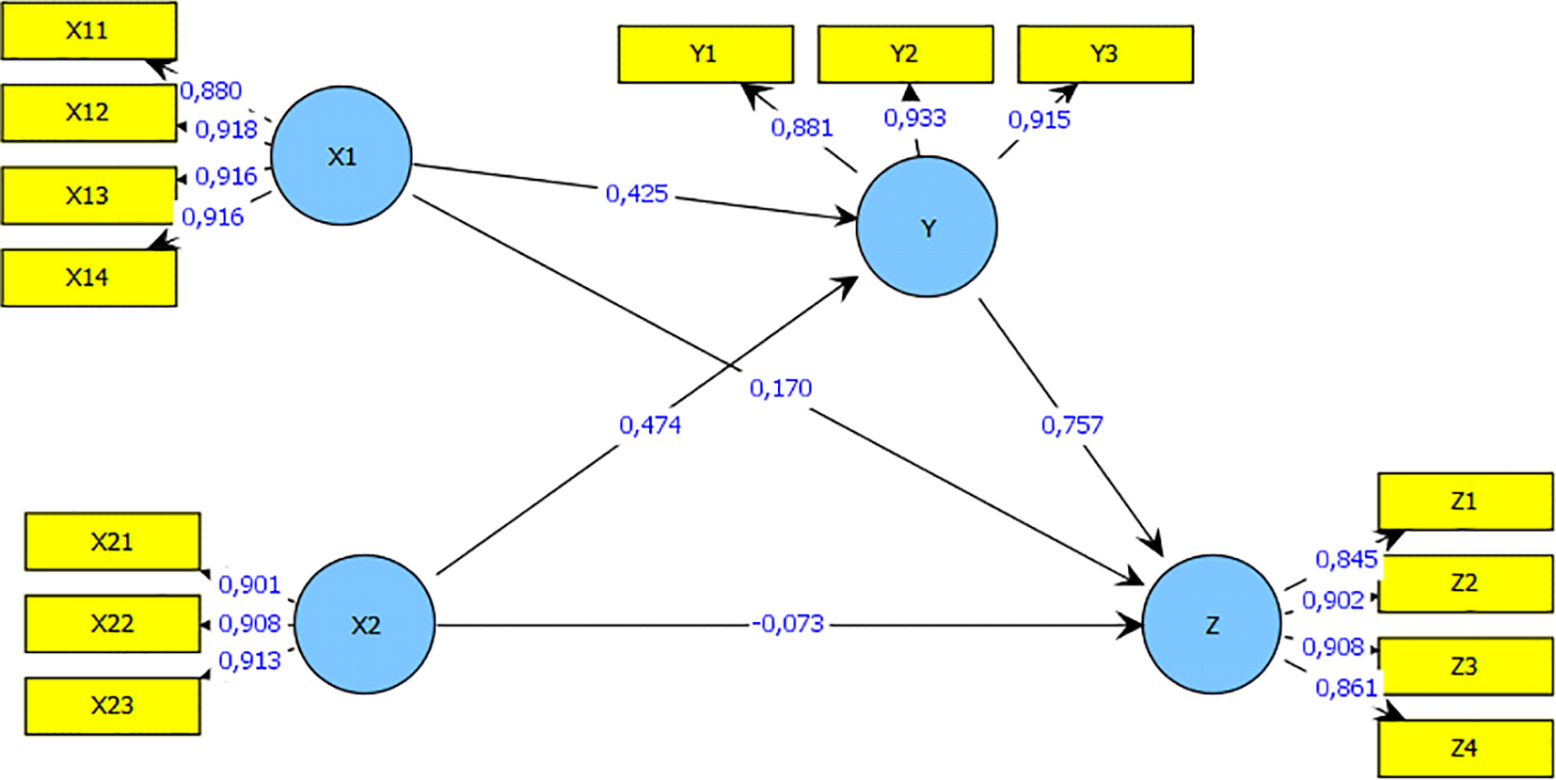
Research model after bootstrapping. Source: Output PLS (2021).

After evaluating the outer and inner models, a bootstrap is carried out to determine the impact of each variable on the performance of SMEs. SE, and Islamic financial literacy on SMEs performance is shown in Figure 1.

According to Table 2, SE has a positive coefficient value of 0, 425, which indicates that it has a significant effect on the performance of SMEs. In addition, the higher the SE, the better the performance of SMEs. Therefore, SE has a significant impact on the performance of SMEs. The Islamic financial literacy variable has a positive coefficient of 0.474. Therefore, both SE and financial literacy have a significant influence on the performance of SMEs.

**Table 2.** Results of the path coefficient test.

	*P*-Value
**Self-efficacy -> SME performance**	*P* = 0.425
**Financial literacy -> SME performance**	*P* = 0.474

Source: Output PLS (2020).

Overall, the majority of respondents indicated that SE increased their confidence in running their business. According to ( Bandura, 2010), high SE has a significant effect on how SMEs handles business-related problems (although there is a reluctance in applying economic theory). Likewise, ( Bandura and Locke, 2003) and ( Ali, Ajmal and Iqbal, 2016) find that goals, SE, and motivation are the three main pillars of SMEs performance. And it means that this research is not in line with ( Vancouver *et al.*, 2002) ( Schmidt and DeShon, 2010) which states that self-efficacy has no impact on business performance.

With regard to social cognitive theory ( Bandura, 2010) found that there is a relationship between SE, motivation, and academic achievement, indicating that SMEs with high SE are generally motivated to improve their business performance. Based on these findings, SE has a positive impact on the performance of SMEs.

Based on the modeling of the least square’s structural equation (PLS-SEM) in this study, Islamic financial literacy has a positive impact on the performance of SMEs. This is in line with his research ( Lusardi and Mitchell, 2017) financial literacy refers to financial knowledge used to achieve prosperity, as well as ( Volpe, Chen and Liu, 2006) suggest that knowledge can be used to manage finances for future prosperity. In addition, this is in line with his research ( ISLAM, no date; Rahayu *et al.*, no date; Suryandani, Nasional and 2019, no date; Anggraeni, 2016; Aribawa, 2016; Literasi *et al.*, 2020). However, SMEs around Unisba still have low Islamic financial liability, making it difficult for them to access capital to Islamic banking. It is within their popularity ( SHOLEH, 2019) people have the financial literacy levels are still low. In this case ( Coad and Tamvada, 2012), shows that lack of knowledge and access to financial resources can hamper a company's ability to achieve its goals and increase its corporate value. Even Djuwita and Yusup ( Djuwita and Yusuf, 2018) in their research stated that Shariah financial literacy has a very significant influence on business development. This will facilitate access to capital and financing to Islamic financial institutions.

Meanwhile, the results of the study found that the influence given the variable self-efficacy (X1), Islamic financial liters (X2) on Business Model Innovation (Y) was 89.9 % and the influence of other variables not included in this study was 10.1 %. While the influence given by the Business Model Innovation Variable (Y) on Business Performance (Z) is 75.7 % and the effect of other variables not included in this study is 24.3%. then the hypothesis is accepted, namely self-efficacy and Islamic financial literacy through business model innovation, **positively** influencing the performance of SMES businesses in Indonesia **simultaneously or partially.**

This is in line with ( Schaltegger and Wagner, 2011) stating business models may need to change (directly or indirectly); namely the need for a better business case trigger effect on the configuration of the business model. Business models are often interpreted as determinants of corporate behavior and thus business opportunities ( Schaltegger, Lüdeke-Freund and Hansen, 2011; Chesbrough, 2010; Schaltegger and Wagner, 2011; Gebauer, Haldimann and Saul, 2017; Aagaard, 2019) who call business models “renewal” business”; Wirtz, Yip, Zott *et al.* ( Yip, 2014; Wirtz, 2011; Zott, Amit and Massa, 2011); that is, the business model in turn influences business performance and operating results (such as cost structures).

The results showed that companies that tried to improve their sustainability performance had to change their business models. Changes in business models can be incremental or radical can turn into determinants (ie limit or support) to successfully create one or many business cases for sustainability (regarding the intensity of modification and innovation of different business models ( Chesbrough, 2010; Gassmann, Enkel and Chesbrough, 2010; Chesbrough, Vanhaverbeke and West, 2014).

### Ethical consideration

This research was approved by the Ethics Licensing Committee of the Universitas Islam Bandung (Protocol number: 660/B.04/Bak-k/XII/2020) after consultation, a letter of approval was given by the researcher to all respondents. Written approval to participate from the Unisba 3IKB Management (Unisba Large Family Mothers Association) was obtained in accordance with document 097/3IKB-K/XII/2020. Written consent to participate was obtained from respondents through the Traders Stall Row. Respondents have given their consent without coercion from anyone. The informed consent was given before the research was conducted by providing a consent form to become a respondent. Respondents who are willing, then they must sign the consent form. Furthermore, to protect the rights and privacy of respondents, all forms of data obtained will be kept confidential.

## Conclusions

This quantitative study examines the effects of self-efficacy and Islamic financial literacy through business model innovation on the performance of SMEs around the Unisba campus. Based on questionnaire surveys and related analysis, self efficacy has a positive effect on the performance of SMEs, because it motivates owners to evaluate their business-related activities. Similarly, Islamic financial literacy has a positive effect on the performance of SMEs. Islamic financial literacy will improve or influence the performance of their business. It's just that the owner of SMEs still lacks an adequate level of Islamic financial knowledge. Product innovation also has a positive effect on SMES performance. So that the novelty of SMES products will make business continuity continue in any condition and situation, especially in pandemic covid-19.

The implication of this finding is that in general the Resource Base View is an important thing in competitive strategies that come from internal organizational sources, especially human resources, so SMEs must improve their SE as best as possible, while integrating their Islamic financial literacy into SMEs to improve overall performance. In addition, MSMEs should ideally implement Islamic financial literacy initiatives, which will not increase the confidence of MSME owners in running their business, but will also improve the performance of their SMEs in achieving long-term competitive advantage.

Further research could include refining Islamic financial literacy resources and data. Limitations of the study include the variety of characteristics of respondents, the sample size is still limited.

## Data availability

Figshare: Dataset of Questionnaire Result from the roles of self efficacy and sharia financial literacy to SMEs performance: business model as intermediate variable. https://doi.org/10.6084/m9.figshare.17082419 ( Srisusilawati, 2021)

This project contains the following basic data:

− Questionnaire results from 40 Indonesian Unisba row stall traders

Figshare: List of questions and description of the questionnaire.

https://doi.org/10.6084/m9.figshare.17082593 ( Srisusilawati, 2021)

This project contains the following additional data:

− A copy of the questionnaire
− Data encoding lock

Data are available under the terms of the Creative Commons Attribution 4.0 International license (CC-BY 4.0).
