# Supplementary material for: The roles of self efficacy and sharia financial literacy to SMES performance: business model as intermediate variable
Source: F1000Res. 2022 Aug 25;10:1310. Originally published 2021 Dec 22. [Version 2] doi: 10.12688/f1000research.76001.2 (PMC9623197; doi:10.12688/f1000research.76001.2)
Supplement: Peer review history

# Peer review: Reviewer response for version 2

**DOI:** 10.5256/f1000research.137565.r153491

**Published:** 2022-10-31

**Authors:** Hafizah Hammad Ahmad Khan

**Affiliations:** 1 Faculty of Business and Management, Universiti Teknologi MARA, Shah Alam, Malaysia

This is very interesting research examining the roles of self-efficacy and sharia financial literacy on SMEs performance. However, a minor revision is required to improve the quality of the paper.

 1. Some abbreviations used in the article was not written in full. Example: MSME, UNISBA should be written in full when it first appears in text before using abbreviations.

 2. Please replace the keyword "literacy Islamic finance" to "sharia financial literacy" and "The performance of SMEs" to "SMEs performance".

 3. In the methodology section, the author should first discuss the research design (questionnaire; sampling, data collection method, etc) then followed by the type of analysis (i.e., PLS-SEM).

 4. This article must be submitted for proofreading.

Is the work clearly and accurately presented and does it cite the current literature?

Yes

If applicable, is the statistical analysis and its interpretation appropriate?

Yes

Are all the source data underlying the results available to ensure full reproducibility?

Yes

Is the study design appropriate and is the work technically sound?

Yes

Are the conclusions drawn adequately supported by the results?

Yes

Are sufficient details of methods and analysis provided to allow replication by others?

Partly

Reviewer Expertise:

Economics; Econometrics; Data analytics

I confirm that I have read this submission and believe that I have an appropriate level of expertise to confirm that it is of an acceptable scientific standard.

# Peer review: Reviewer response for version 2

**DOI:** 10.5256/f1000research.137565.r148543

**Published:** 2022-10-06

**Authors:** Bambang Tjahjadi

**Affiliations:** 1 Faculty of Economics and Business, Airlangga University, Surabaya, Indonesia

She paper has been improved significantly. So, I recommend for publication.

Is the work clearly and accurately presented and does it cite the current literature?

Partly

If applicable, is the statistical analysis and its interpretation appropriate?

Partly

Are all the source data underlying the results available to ensure full reproducibility?

Partly

Is the study design appropriate and is the work technically sound?

Partly

Are the conclusions drawn adequately supported by the results?

Partly

Are sufficient details of methods and analysis provided to allow replication by others?

Partly

Reviewer Expertise:

business management and accounting

I confirm that I have read this submission and believe that I have an appropriate level of expertise to confirm that it is of an acceptable scientific standard.

# Response

**Published:** 2022-10-07

**Authors:** Popon Srisusilawati

**Affiliations:** Universitas Islam Bandung, Indonesia

Thank you very much.

# Peer review: Reviewer response for version 1

**DOI:** 10.5256/f1000research.79950.r118595

**Published:** 2022-04-11

**Authors:** Bambang Tjahjadi

**Affiliations:** 1 Faculty of Economics and Business, Airlangga University, Surabaya, Indonesia

Introduction:

1. The research gap is not clearly stated.
2. The justification for choosing the mediating variable of business model is needed.
3. The sample size is too small for the study of SMEs.
4. The reasons for choosing street vendors in Bandung is not clearly described.

Literature Review:

1. The use of RBV is not specifically explained, what is its relevance for the current study.
2. No hypotheses are developed for this study.

Method:

1. Sample size equals to population (40) vendors (SMEs) is insufficient for SMEs study.
2. How to deal with common method variance (CMV) is absent in this study.

Results and Discussions:

1. The interpretation of statistical attributes need correction (p-value is not the same with beta coefficient).
2. Discussions need elaborations, especially the relevance of the findings (using 40 samples) to the wider audience.
3. It is difficult to discuss the findings without the development of hypotheses.

The implications of this study needs further elaboration, to SMEs and their stakeholders (in both discussion and conclusion).

 Language:

- A thorough proofreading of the manuscript is recommended.

Is the work clearly and accurately presented and does it cite the current literature?

Partly

If applicable, is the statistical analysis and its interpretation appropriate?

Partly

Are all the source data underlying the results available to ensure full reproducibility?

Partly

Is the study design appropriate and is the work technically sound?

Partly

Are the conclusions drawn adequately supported by the results?

Partly

Are sufficient details of methods and analysis provided to allow replication by others?

Partly

Reviewer Expertise:

business management and accounting

I confirm that I have read this submission and believe that I have an appropriate level of expertise to state that I do not consider it to be of an acceptable scientific standard, for reasons outlined above.

# Response

**Published:** 2022-04-20

**Authors:** Popon Srisusilawati

**Affiliations:** Universitas Islam Bandung, Indonesia

Thanks for the feedback and review.
